# A Novel Signal Separation and De-Noising Technique for Doppler Radar Vital Signal Detection

**DOI:** 10.3390/s19214751

**Published:** 2019-11-01

**Authors:** Xiaoling Li, Bin Liu, Yang Liu, Jiawei Li, Jiarui Lai, Ziming Zheng

**Affiliations:** School of Mechanical Engineering, Xi’an Jiaotong University, Xi’an 710049, Chinaxjtu_liuyang@stu.xjtu.edu.cn (Y.L.); lijiawei@stu.xjtu.edu.cn (J.L.); ljr285@stu.xjtu.edu.cn (J.L.); zmzheng121719@stu.xjtu.edu.cn (Z.Z.)

**Keywords:** doppler radar, vital signal, separation and de-noising, ICEEMDAN, sample entropy, wavelet threshold

## Abstract

Doppler radar for monitoring vital signals is an emerging tool, and how to remove the noise during the detection process and reconstruct the accurate respiration and heartbeat signals are hot issues in current research. In this paper, a novel radar vital signal separation and de-noising technique based on improved complete ensemble empirical mode decomposition with adaptive noise (ICEEMDAN), sample entropy (SampEn), and wavelet threshold is proposed. First, the noisy radar signal was decomposed into a series of intrinsic mode functions (IMFs) using ICEEMDAN. Then, each IMF was analyzed using SampEn to find out the first few IMFs containing noise, and these IMFs were de-noised using the wavelet threshold. Finally, in order to extract accurate vital signals, spectrum analysis and Kullback–Leible (KL) divergence calculations were performed on all IMFs, and appropriate IMFs were selected to reconstruct respiration and heartbeat signals. Moreover, as far as we know, there is almost no previous research on radar vital signal de-noising based on the proposed technique. The effectiveness of the algorithm was verified using simulated and measured experiments. The results show that the proposed algorithm could effectively reduce the noise and was superior to the existing de-noising technologies, which is beneficial for extracting more accurate vital signals.

## 1. Introduction

In recent years, non-contact vital signal detection based on Doppler radar has attracted wide attention [1,2,3,4]. Radar has unique advantages in vital signal detection. Remote monitoring can be performed without direct contact with the subject, and is not susceptible to environmental factors, such as weather, temperature, and light [5]. Radar waves have a strong penetrating ability, which is of great significance for long-term physiological monitoring in special occasions. Especially in the health monitoring and sleep monitoring fields, radar plays an important role. In the field of sleep monitoring, different sleep states are obtained via feature extraction and machine learning classification of the separated radar signals [6]. At the same time, some scholars have studied the accuracy of the classification of sleep states in different postures and different orientations [7]. Radar is also a promising method to assess psychophysiological conditions by detecting the pressure level [8]. In terms of radar structure, many of the radar systems of the past are now in some devices for integrated circuit chips and printed circuit boards. Typical radar systems currently include unmodulated radar, FMCW radar, and hybrid mode radar [9]. Although the analog circuit in the radar system can remove some noise, it will still receive interference signals caused by other objects and a human body’s own jittering within a similar distance. Moreover, heartbeat signals are likely to be submerged in respiratory harmonics, which makes it difficult to extract heartbeat signals [10]. Therefore, an appropriate de-noising algorithm is needed to remove noise interference from noisy radar signals and extract pure respiratory and heartbeat signals.

In the past few years, the traditional radar vital signal processing methods have mostly used filtering to achieve the removal of noise and separating the respiratory and heartbeat signals [11,12]. However, due to the limitation of the passband range, only noise outside the vital signal band can be removed. In-band noise and respiratory harmonic interference cannot be removed, and due to the attenuation of the stop band, a relatively accurate vital signal cannot be obtained. At the same time, some scholars have proposed to extract periodic vital signals from irregular noise signals using an adaptive noise cancellation algorithm [13,14]. However, due to the limitation of the reference signal input, the adaptive signal filtering requires two-signal inputs, i.e., two radars, which brings difficulties to the experimental operation and reduces the accuracy. In order to solve this problem, an adaptive linear enhancement method is adopted, and the delayed signal of the original signal is used as a reference input, which greatly reduces the complexity of the experiment [15,16]. Some scholars have also proposed a peak detection algorithm to extract vital signals. Due to the working characteristics of the radar, the vital signs obtained by the radar sensor are not as obvious as the traditional ECG signals. Even if this algorithm can obtain the heart rate information, due to the limitation of the algorithm itself, the time–frequency domain analysis cannot be performed. Therefore, it cannot reflect time-varying characteristics of physiological signals and other detailed characteristics [17]. On the issue of de-noising, some studies have applied wavelet de-noising to simulate chest wall motion. The radar signal is de-noised by adding different noise signals through simulation to enhance the adaptability of the algorithm [18]. However, the separation of noise in the heartbeat signal is still not possible, and the signal cannot be processed on different time scales. In addition, there are the adaptive harmonic comb-filter algorithm [19], extended Kalman filter [20], and independent component analysis algorithm [21] for environmental, system de-noising, and clutter suppression, but the respiratory signal harmonic problem and heartbeat signal noise removal effect is not ideal.

Empirical mode decomposition (EMD) is an adaptive signal decomposition algorithm for non-linear and non-stationary signals [22]. It was proposed by Huang et al. in 1998. Compared with the traditional signal processing algorithm, not only does EMD break through the limitation of the Fourier transform, but it also does not have the problem of preselecting a wavelet basis function like a wavelet transform does. It has a good time resolution and self-adaptability and can reconstruct the signal perfectly.

The local characteristics of EMD may have oscillations of different scales in one mode or similar scale oscillations in different modes. This situation produces the problem of “mode mixing.” In order to solve this problem, a new ensemble empirical mode decomposition (EEMD) method is proposed [23]. The method decomposes an ensemble of noisy copies of the original signal and gets the result via averaging. However, the EEMD algorithm still has the problem that signals plus different noise will produce a different number of modes. A complete ensemble empirical mode decomposition with adaptive noise (CEEMDAN) is an important improvement to EEMD [24]. Its reconstruction error is almost negligible. Still, CEEMDAN still needs some necessary improvements. There are some residual noises in its mode, and there are some “spurious” modes in the early stages of decomposition [25]. Therefore, we adopt the improved CEEMDAN (ICEEMDAN).

ICEEMDAN effectively solves the above two problems. It was proposed by Colominas et al. in 2014 [26]. Due to the non-stationary nature of vital signals and the distribution of raw respiratory and heartbeat signals on different time scales, this algorithm is particularly suitable for the processing of biomedical signals.

In this paper, a novel signal separation and de-noising method based on ICEEMDAN, sample entropy (SampEn), and wavelet threshold for radar vital signals is proposed. However, as far as we know, there is almost no previous research on radar vital signal de-noising based the proposed technique. Compared with the other existing algorithm, the proposed algorithm can de-noise the signals separately at different time scales. It has a better de-noising and separation effect, and can retain the details of the signal to the greatest extent.

## 2. Model and Measurement Setup

### 2.1. The Vital Signal Model Based on Doppler Radar Measurements

**Figure 1:**
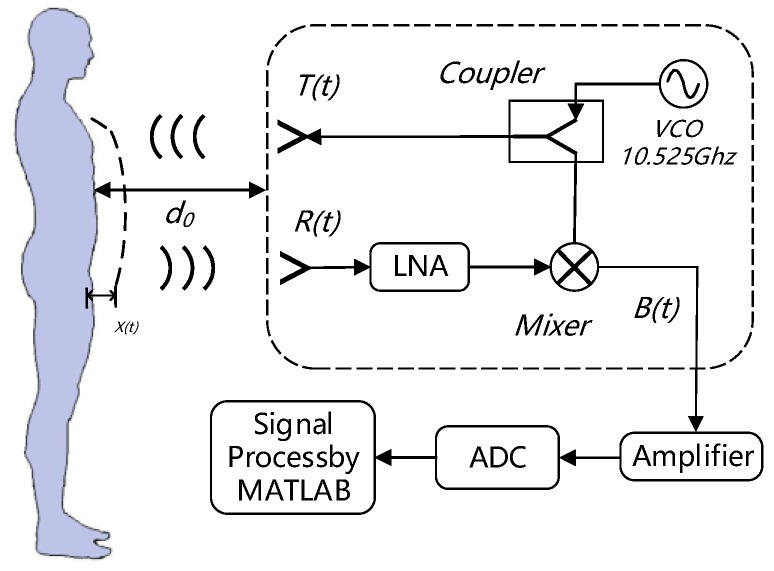
The theoretical basis for detecting respiratory and heartbeat signals using CW radar.

The basic model of using continuous wave (CW) radar to monitor human vital signals is shown in Figure 1.

For the frequency selection of the radar module, the higher frequency radar has a higher resolution and smaller volume and its transmission capability is enhanced, but the energy of the reflected signal is weak. Therefore, considering the need for transmission capacity and volume, we selected an X-band (10.525 GHz) radar module.

In a CW radar system, the transmitting antenna emits an X-band signal, and the reflected wave is received by the receiving antenna. When an electromagnetic wave signal reaches a target and is reflected, frequency modulation occurs due to the motion of the target. Generally, the Doppler shift in frequency is given as:

(1) $$f_{d}(t)=\frac{2fv(t)}{c}=\frac{2v(t)}{\lambda }$$

where *v*(*t*) is the velocity of the target, *λ* is the wavelength of the transmitted signal, *f* is the frequency of the transmitted signal, and *c* is velocity of the propagating wave.

Suppose the target is at a distance *d*_0_, with a time-varying chest wall displacement *x(t)*, and the distance between the target and the transceiver is *d*(*t*) = *d*_0_ + *x*(*t*). When the target of the radar detection is the chest cavity, the Doppler frequency shift can be represented in the form of a non-linear phase signal *θ*(*t*) given as Equation (2) and the transmitted signal is given as Equation (3):

(2) $$\theta (t)=2\pi \times \frac{2x(t)}{\lambda }=\frac{4\pi x(t)}{\lambda }$$

(3) $$T\left(t\right)=A_{T}\cos\left(2\pi ft+\phi \left(t\right)\right)$$

where *T*(*t*) is the transmitted signal, *A_T_* is the amplitude of the signal, and *ϕ*(*t*) is the initial phase noise of the oscillator. The *R*(*t*) obtained by the receiver is the delayed signal of the transmitted signal, and *t_d_* is the delay time generated during the signal propagation:

(4) $$t_{d}=\frac{2d\left(t-\frac{d(t)}{c}\right)}{c}=\frac{2\left(d_{0}+x(t-\frac{d(t)}{c})\right)}{c}$$

(5) $$\begin{array}{l} R\left(t\right)=A_{R}\cos\left(2\pi f\left(t-t_{d}\right)+\phi \left(t-t_{d}\right)+\theta_{0}\right)\\ =A_{R}\cos\left(2\pi ft-\frac{4\pi d_{0}}{\lambda }-\frac{4\pi x(t-\frac{d(t)}{c})}{\lambda }+\phi \left(t-\frac{2d_{0}}{c}-\frac{2x(t-\frac{d(t)}{c})}{c}\right)+\theta_{0}\right)\\ \approx A_{R}\cos\left(2\pi ft-\frac{4\pi d_{0}}{\lambda }-\frac{4\pi x(t)}{\lambda }+\phi \left(t-\frac{2d_{0}}{c}\right)+\theta_{0}\right) \end{array}$$

where *θ*_0_ is the constant phase shift generated during the reflection of the target surface, which is close to 180°. As the signal is also transmitted while the chest wall is moving, the distance between the antenna and the chest wall at the time of reflection is denoted as *d* (*t − d*(*t*)*/c*).

After the *R*(*t*) passes through the low noise amplifier (LNA), it is converted to a baseband signal *B*(*t*) via a mixer, and the mixer mixes the received signal and the copy of the transmitted signal generated by the voltage-controlled oscillator (VCO):

(6) $$B\left(t\right)\approx A_{B}\cos\left(\theta +\theta \left(t\right)+\Delta \phi \left(t\right)\right)=A_{B}\cos\left(\frac{4\pi d_{0}}{\lambda }-\theta_{0}+\frac{4\pi x\left(t\right)}{\lambda }+\Delta \phi \left(t\right)\right)$$

where $\theta =4\pi d_{0}/\lambda -\theta_{0}$ is the constant phase shift related to the parameters of the receiver itself and depend on the nominal distance to the target, and Δ*ϕ*(*t*) is the residual phase noise.

It can be seen from the baseband signal that the change in signal is only related to the initial distance *d*_0_ and the Doppler phase shift, while the Doppler phase shift is only related to the time-varying chest wall displacement *x*(*t*). Therefore, we can obtain the chest wall displacement of the human body through the baseband signal.

### 2.2. Measurement Setup

A commercial CW Doppler radar HB100 (ST Engineering Ltd, Singapore) combined with a custom data acquisition system was chosen. The microwave band of 10.525 GHz has a good directivity and is easily attenuated during transmission and it will not cause harm to the human body because it works in a safe range.

The millivolt baseband signal from the transceiver was first subjected to amplification filtering processing of the analog signal before being processed by the digital signal. Therefore, we designed a set of targeted analog filter amplifier circuits. After the analog circuit filtering and amplifier processes, the Arduino’s 10-bit AD module (Arduino UNO R3) was used to convert the analog signal into a digital signal, and then the serial port data was read by the host computer for display and data processing.

**Figure 2:**
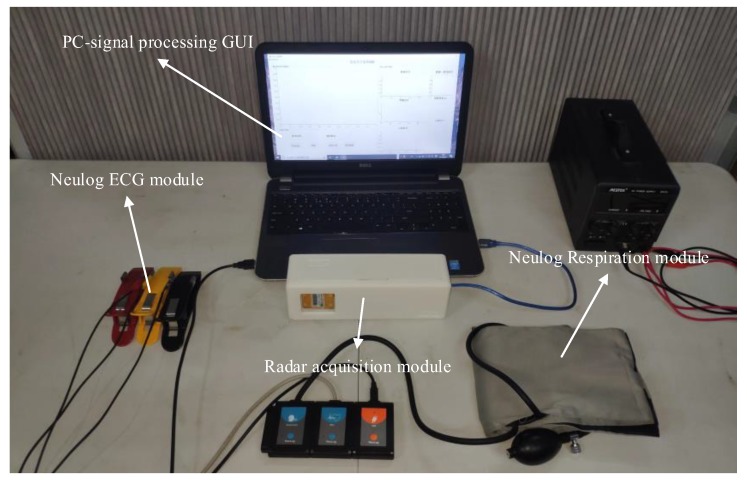
Measurement setup: Doppler radar module, Neulog ECG sensor, Neulog RSP sensor, and PC. GUI—graphical user interface.

At the same time, we aimed to prove the effectiveness of the radar acquisition of physiological signals; therefore, we used Neulog’s Electrocardiogram logger sensor NUL-218 and Respiration Monitor Belt logger sensor NUL-236 (Neulog, Israel) to collect reference ECG signals and reference respiratory signals. The measurement setup is depicted in Figure 2.

## 3. Methods

### 3.1. ICEEMDAN

ICEEMDAN—as an improved algorithm of EMD, EEMD, and CEEMDAN—effectively solves the shortcomings of other methods, and is especially suitable for analyzing biological signals.

EEMD has the problem of producing different mode numbers when different noises are added. CEEMDAN improves this by adding paired noise (one positive and one negative) to the original signal. Therefore, CEEMDAN overcomes the issues of inconsistent modal numbers during the decomposition process. However, EMD and CEEMDAN behave similarly in improving the mode-mixing ability. CEEMDAN still has two major problems: the presence of residual noise in the modes and the existence of spurious modes. This makes it more difficult to de-noise a signal that is already noisy. We tried to improve this status with ICEEMDAN. The overall algorithm was as follows:

*E_k_*(*∙*) is specified as the *k*th IMF component of the EMD decomposition, *M*(*∙*) is the local mean of the signal, <·> is the overall average of the signal, x(i)=x+βEi(w(i)) is a noisy signal, and *w*(*i*) is the added Gaussian white noise.

1. Decompose the noisy signal $x(i)=x+\beta_{0}E_{1}(w(i))$ using EMD to obtain the first residue and the first IMF:
  (7) $$r_{1}=<M(x(i))>$$
  (8) $$IMF_{1}=x-r_{1}$$
2. Take the local average of $r_{1}+\beta_{1}E_{2}(w(i))$ as the second residue and define the second mode:
  (9) $$IMF_{2}=r_{1}-<M(r_{1}+\beta_{1}E_{2}(w(i)))>$$
3. Calculate the *k*th residue k=3,…,K:
  (10) $$r_{k}=<M(r_{k-1}+\beta_{k-1}E_{k}(w(i)))>$$
4. Compute the *k*th mode:
  (11) $$IMF_{K}=r_{k-1}-r_{k}$$
5. Repeat steps 3 and 4 until all IMFs are extracted. If the residual obtained in step 3 does not satisfy the condition of further EMD decomposition, terminate the calculation process.

We chose the ICEEMDAN algorithm for the following reasons:

- ICEEMDAN has a better decomposition performance, effectively solving the problem of mode mixing, inconsistent IMF number with different noise, and partial residual noise.
- ICEEMDAN is suitable for decomposing non-linear, non-stationary signals. In theory, the essence of ICEEMDAN decomposition is to smooth out a sequence. The result is the decomposition of the fluctuations or trends of different time scales in the signal to produce a series of data sequences with different feature scales. The radar signal containing the vital signal is completely in accordance with the characteristics of the algorithm because the vital signals have a fixed range of time scales.
- The ICEEMDAN algorithm is adaptive and can be decomposed from different time scales according to the characteristics of the signal without the need for a basis function.

### 3.2. Sample Entropy

Sample entropy (SampEn) is an improved method of approximate entropy (ApEn), which can be used to measure the complexity of a time series. It was proposed by Richman and Moorman in 2000 [27]. This method is mainly used to analyze noisy data sets encountered in cardiovascular and other biological studies. It can be used to evaluate the complexity of physiological time series and diagnose a pathological status. The specific algorithm is as follows:

Assume that there is a time series $Xi=\{x_{1},x_{2},x_{3},\ldots ,x_{N}\}$; its length is *N*.

1. Define the algorithm-related parameters, where *m* is the length of the sequence to be compared and *r* is the tolerance to accept the match and consider the M-dimensional vector group ${\{x}_{m}{(1),x}_{m}(2),\ldots {,x}_{m}\left(N-m+1)\right\}$.
  (12) $$X_{m}(i)=\{x(i),x(i+1),\ldots ,x(i+m-1)\},1\leq i\leq N-m+1$$
2. Suppose that the distance $d[X_{m}(i),X_{m}(j)]$ between a vector x(i+k) and x(j+k) is the maximum value of the absolute value of the difference between the corresponding elements of a vector. That is:
  (13) $$d[X_{m}(i),X_{m}(j)]=\max_{k=0,1,2,\ldots ,m-1}(\left|x(i+k)-x(j+k)\right|)$$
3. Given the tolerance r(r>0), the number of $d[X_{m}(i),X_{m}(j)]<r$ is counted for each i value, and is denoted as $B_{i}$. Calculate the ratio of $B_{i}$ to the total number of *N* − *m* distances, which is denoted as $B_{i}^{m}(r)$. Then, find $B_{i}^{m}(r)$ for the average of all i values, which is denoted as $B^{m}(r)$:
  (14) $$B_{i}^{m}(r)=\frac{1}{N-m-1}B_{i}$$
  (15) $$B^{m}(r)={\left(N-m+1\right)}^{-1}\sum_{i=1}^{N-m+1}B_{i}^{m}(r)$$
4. Increase the dimension to *m* + 1, and calculate the number of $X_{m+1}(i)$ and $X_{m+1}(j)$ less than or equal to *r*, which is denoted as $A_{i}$. Define $A_{i}^{m}(r)$ as:
  (16) $$A_{i}^{m}(r)=\frac{1}{N-m-1}A_{i}$$
  (17) $$A^{m}(r)=\frac{1}{N-m}\sum_{i=1}^{N-m}A_{i}^{m}(r)$$
  According to the above analysis, $B_{m}(r)$ is the probability that two sequences match *m* points under the tolerance *r*. $A^{m}(r)$ is the probability that two sequences match *m* + 1 points.
5. Then, the SampEn of this time series can be defined as:
  (18) $$SampEn(m,r)=\lim\{-\ln[\frac{A^{m}(r)}{B^{m}(r)}]\}$$
  when the length of the time series is *N*, the estimated value of the sample entropy is:
  (19) $$SampEn(m,r,N)=-\ln[\frac{A^{m}(r)}{B^{m}(r)}]$$

The dimension *m* and the threshold *r* are the two main parameters of the SampEn. According to the research results of Pincus, *m* = 1 *or m* = 2, *r* = 0.1 *Std* can obtain the sample entropy with more reasonable statistical characteristics. In this paper, the parameter values were *m* = 2 and *r* = 0.2, and the algorithm was chosen for the following reasons:

- The SampEn analyzes the complexity of the time series by measuring the size of the new pattern in the signal. The required target signal is a periodic signal, and the more noise it contains, the more complex the signal.
- The SampEn should be a useful tool in studies of the dynamics of a human physiological signal.

### 3.3. Improved Wavelet Threshold

Wavelet transform is a multi-scale signal analysis method, and its excellent de-noising effect is very popular. The process of wavelet de-noising can be divided into the following steps:

1. The appropriate wavelet base and the number of decomposition layers are selected to perform wavelet decomposition on the noisy signals to obtain wavelet coefficients corresponding to different decomposition layers.
2. Select the appropriate threshold function and threshold to sift the corresponding wavelet coefficients.
3. Perform an inverse transformation on the sifted wavelet coefficients to reconstruct the de-noised signal.

The selection of the wavelet threshold function is the key part of de-noising. The soft and hard threshold functions proposed by Donoho et al. [28] have been widely used in practice. Combining the characteristics of soft threshold and hard threshold functions, this paper uses an improved threshold function [29] to estimate wavelet coefficients:

(20) $${\overset{\wedge }{d}}_{j}=\{\begin{array}{c} \mathrm{sgn}(d_{j})(\left|d_{j}\right|-\beta^{\left(T_{j}-\left|d_{j}\right|\right)}T_{j})\\ 0 \end{array}\begin{array}{c} ,\left|d_{j}\right|\geq T_{j}\\ ,\left|d_{j}\right|<T_{j} \end{array}$$

where β>1 and β∈R. In the above formula, when β→∞, this is equivalent to a hard threshold function, and when β→0, this is equivalent to a soft threshold function. This reflects the adaptability of the improved threshold function. The threshold value is chosen using:

(21) $$T_{j}=\sigma \sqrt{2\log\left\|d_{j}\right\|}/\log(j+1)$$

where $\overset{\wedge }{d_{j}}$ represents the wavelet coefficient.

Therefore, the improved wavelet threshold de-noising method can be seen as a compromise between the soft threshold and the hard threshold method. The appropriate β value can be selected via analysis through trial and error to meet the de-noising requirements of the radar signal. Here, we chose the β value of 25 based on previous experience.

### 3.4. Kullback–Leible (KL) Divergence

KL divergence is also known as relative entropy. To some extent, entropy can measure the distance between two random variables. KL divergence is an asymmetry measure of the difference between two probability distributions *P* and *Q*. Assuming that *P* and *Q* are the two probability distributions of *x*, the relative entropy of *P* to *Q* is:

(22) $$D(p∥q)=\sum_{i=1}^{n}p(x)\log\frac{p(x)}{q(x)}$$

The KL divergence measures the distance between two random distributions. When the two random distributions are the same, their relative entropy is zero. When the difference between the two random distributions increases, their relative entropy also increases. Therefore, the breath and heartbeat signals can be discerned by calculating the KL divergence value between each IMF and the original signal.

## 4. Separation and De-Noising Technique

### 4.1. The Steps of the Separation and De-Noising Technique

The raw radar signal is often inevitably accompanied by noise during the acquisition process. Some noise comes from the body movement of the subject and some noise comes from the background noise of the experimental environment. The chest wall motion caused by the heartbeat motion is very weak, generally one-fifth of the respiratory signal. Therefore, the heartbeat signal will not only be buried in the respiratory harmonics, but also be interfered with by various noises, which will create difficulties regarding the extraction of an accurate heartbeat signal.

**Figure 3:**
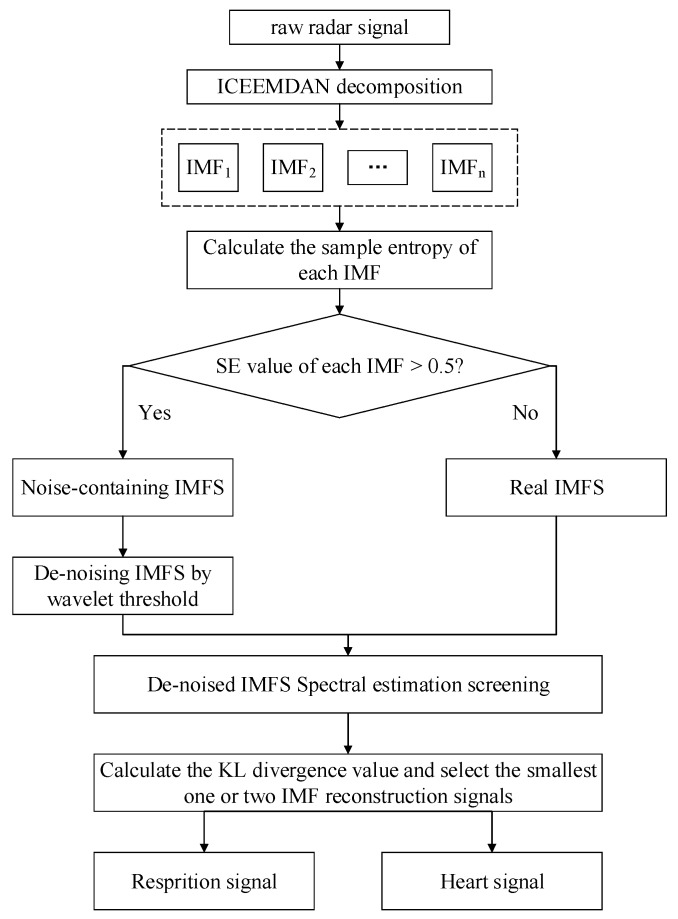
The flow chart of radar signal processing.

To remove the noise present in the signal, the respiratory and heartbeat signals are separated from the original radar signal. The algorithm block diagram of the signal processing is shown in Figure 3. The specific process is summarized as follows:

1. The raw signal is preprocessed, including removing the first and last invalid signals and de-trending. The preprocessed radar signal is decomposed using ICEEMDAN, retrieving a lot of IMFs, including noise containing IMFs and real IMFs, in the process.
2. The sample entropy of each IMF is calculated to characterize the regularity and complexity of each IMF.
3. The noise-containing IMF is identified using SampEn. If the sample entropy of the IMF is greater than 0.5, it can be considered as a noise-containing IMF; otherwise, it is a real IMF. The threshold of 0.5 is an empirical value. It can distinguish whether the sample entropy is noisy or not under the premise that the sample entropy is generally declining.
4. The noise-containing IMF is de-noised using a wavelet threshold. We used the improved wavelet threshold function for de-noising the noise-containing IMFs, and decomposition levels were sym6 and sym4.
5. By estimating the frequency spectrum of the de-noised IMFs and the real IMFs, the IMFs with the frequency range of 0.2–0.6 Hz and 0.9–1.5 Hz were selected as the potential respiratory signal and heartbeat signal.
6. The KL divergence value of the potential respiratory signal is calculated and the first two IMF reconstructed respiratory signals with the largest KL divergence value are selected. The potential heartbeat signal receives the same action. If the number of IMFs is less than 2, it is directly used as the target signal.

### 4.2. Simulated Signal Processing

We first constructed the simulated signal to verify the effectiveness of the algorithm. The radar demodulation signal characterizes the reciprocating motion of the human chest wall. Its active components include respiratory signals and heartbeat signals. We constructed a respiratory signal and a heartbeat signal with the fundamental frequencies of 0.28 Hz and 1.28 Hz, respectively. Then, we added Gaussian noise of 0 dB, 5 dB, and 10 dB signal-to-noise ratio (*SNR*). The sampling time and data length of the data were 60 s and 1000, respectively.

**Figure 4:**
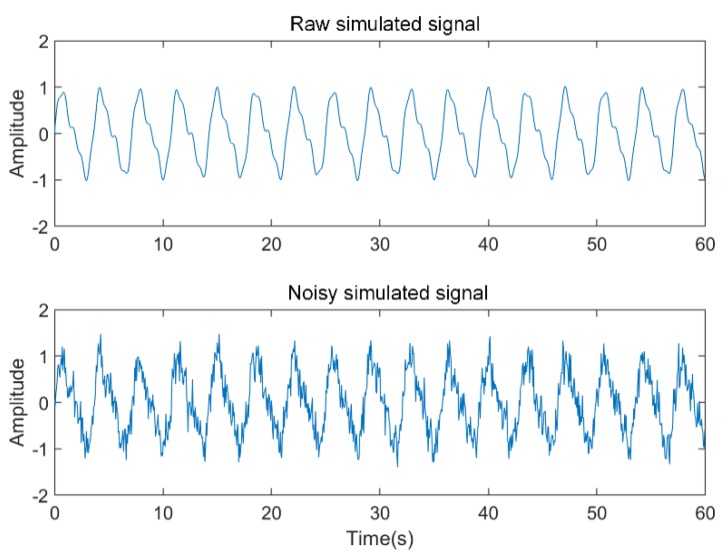
The raw simulated signal and noisy simulated signal.

**Figure 5:**
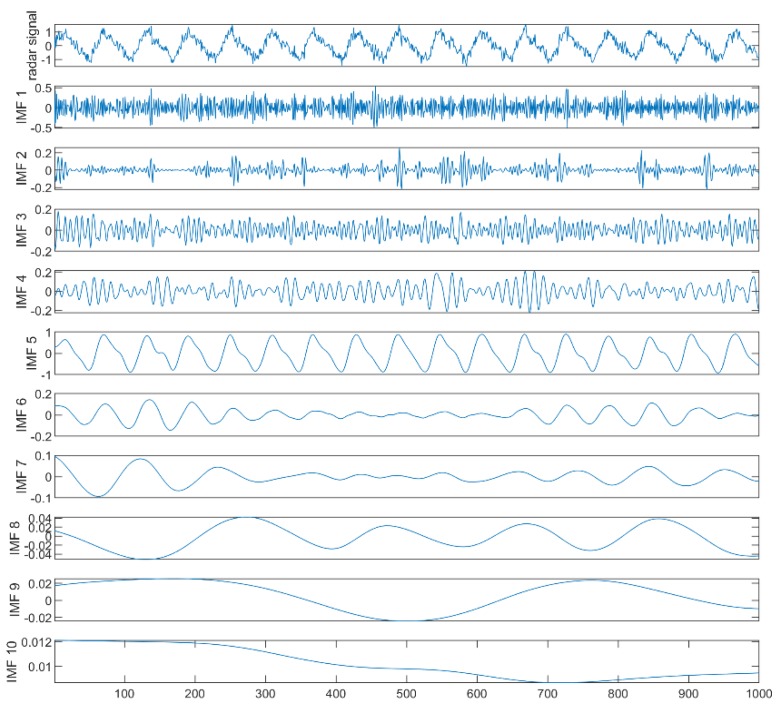
The decomposition result of the pre-processed signal.

Take the signal of 5 dB *SNR* as an example. The raw waveform of the simulated signal and the time domain waveform of the 5 dB *SNR* signal are shown in Figure 4. The result of the signal decomposition is shown in Figure 5. The noisy simulated signal was decomposed using ICEEMDAN. A series of IMFs with high frequency to low frequency are obtained. In general, the higher the frequency of the IMF, the more noise it contained. Then, the IMF containing noise was identified by calculating the sample entropy for each IMF as shown in Table 1.

**Figure 6:**
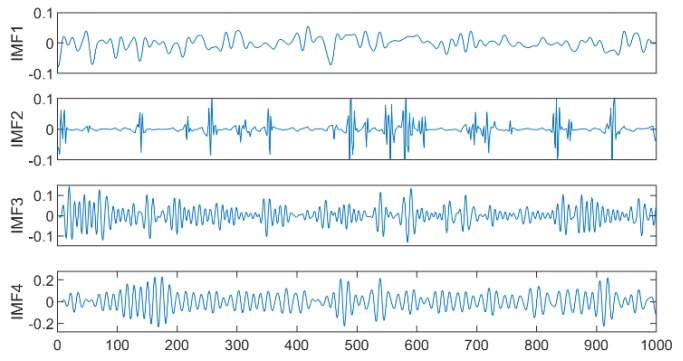
The IMFs de-noised results using an improved wavelet threshold.

Table 1 shows that the first four IMFs were determined to be noisy IMFs according to the previously set threshold of 0.5. The improved wavelet threshold de-noising for the first four IMFs was undertaken, and Figure 6 shows the de-noised IMFs using the improved wavelet threshold. Then, the respiratory and heartbeat signals were distinguished using spectral estimation, and the KL divergence was calculated to screen out the IMFs with the highest correlation between the respiration and heartbeat for respiration and heartbeat signal reconstruction.

**Figure 7:**
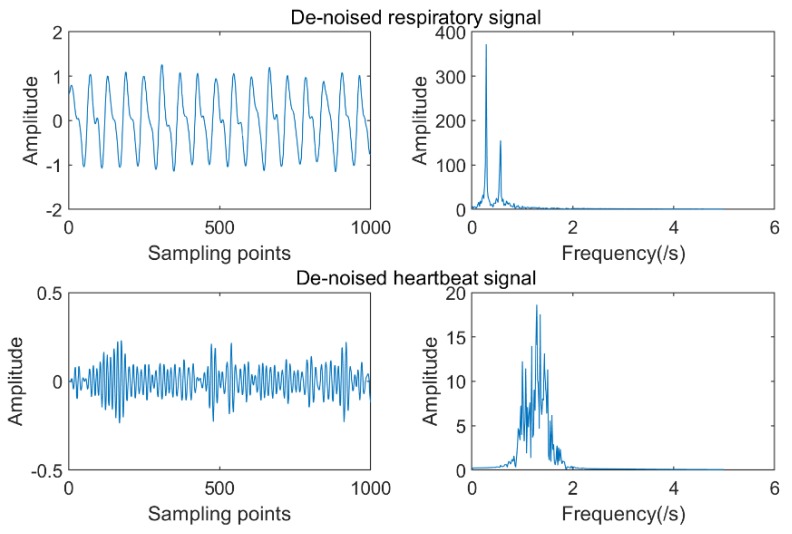
De-noised respiratory and heartbeat waveforms and spectrograms.

The IMFs conforming to the heartbeat signal spectrum (0.9–1.5 Hz) were IMF3 and IMF4, and for the respiratory signal spectrum (0.2–0.6 Hz), there were IMF5 and IMF6. Calculation results for the KL divergence value of the corresponding IMF separately is shown in Table 2, and the smallest one or two IMF reconstruction signals that met the spectral estimation conditions were selected. The final heartbeat signal was reconstructed using IMF4 and the respiratory signal was reconstructed using IMF5 and IMF6, as shown in Figure 7.

## 5. Results and Discussion

### 5.1. Results of the Simulated Signal

**Figure 8:**
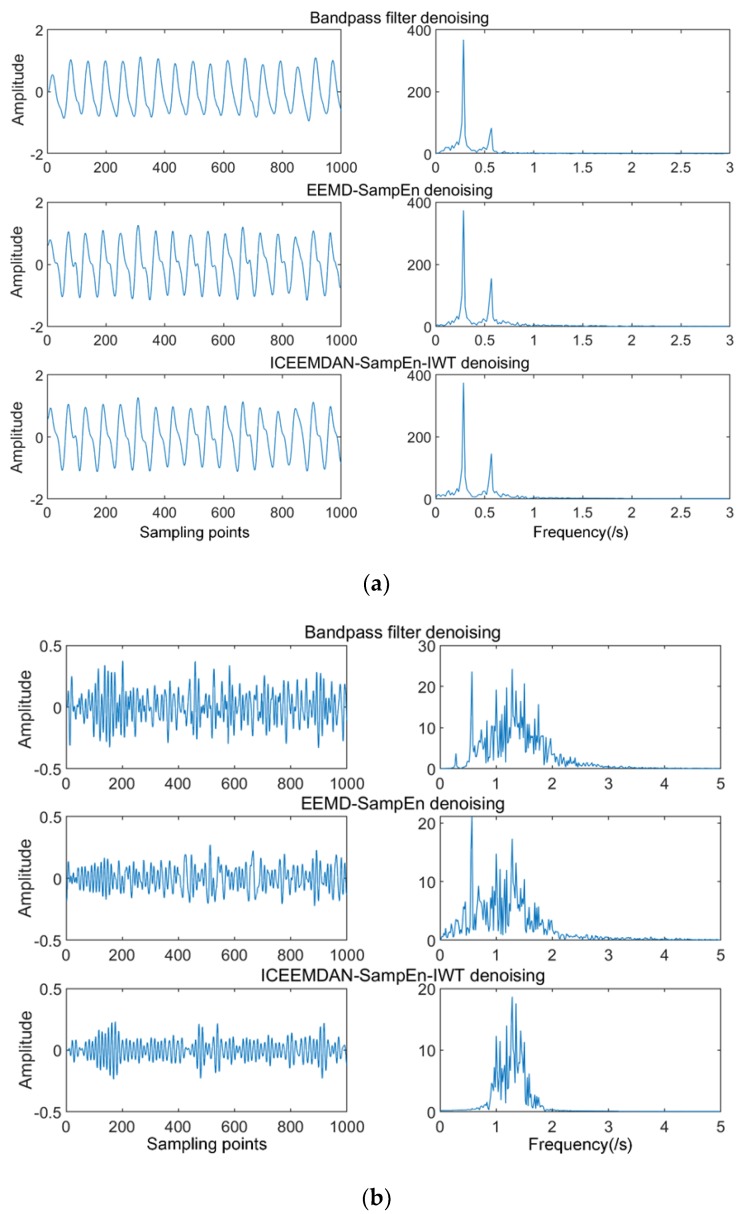
(**a**) Respiratory waveforms and spectrograms using three algorithms (simulated signal). (**b**) Heartbeat waveforms and spectrograms using three algorithms (simulated signal).

We de-noised the signal of the 5 dB *SNR* using the proposed algorithm in the previous section. For comparison purposes, we also used bandpass filtering and the EEMD-SE algorithm to process the same signal. The de-noising results and the waveform and spectrogram are given in Figure 8. By analogy, we also applied three algorithms to the signals of 0 dB *SNR* and 10 dB *SNR*, and compared the performance of signals with different degrees of noise under the three algorithms. Performance indicators under different intensity noises are shown in Table 3.

**Figure 9:**
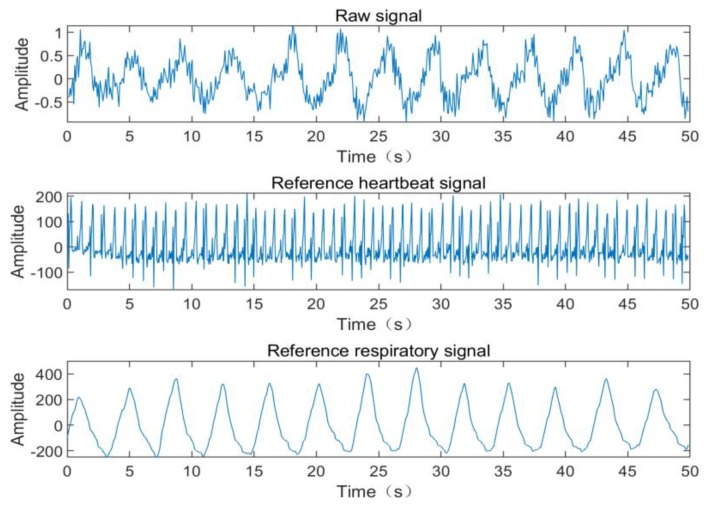
Measured noisy radar signal and reference signal.

As can be seen from Figure 8, the separation de-noising method using ICEMDAN-SampEn-IWT had the most concentrated spectral peaks and contained less noise in the spectrum. Although the bandpass filtering in Figure 9a was more prominent when extracting respiratory signals, our proposed method retained more detailed features, such as differences in exhalation and inhalation processes. To quantify the superiority of the proposed algorithm, the signal-to-noise ratio (*SNR*) and mean square error (*MSE*) were used as indicators of de-noising performance. *SNR* reflects the ratio of signal to noise. *MSE* defines the energy of the noise signal. The equations for *SNR* and *MSE* were redefined as:

(23) $$SNR=10\log_{10}\left(\frac{\sum_{f_{major-B/2}}^{f_{major+B/2}}l^{2}(f)}{\sum_{0}^{N}l^{2}(f)-\sum_{f_{major-B/2}}^{f_{major+B/2}}l^{2}(f)}\right)$$

(24) $$MSE=\frac{1}{N}{\left(\sum_{0}^{N}l(f)-\sum_{f_{major-B/2}}^{f_{major+B/2}}l(f)\right)}^{2}$$

where $\sum_{f_{major-B/2}}^{f_{major+B/2}}l^{2}(f)$ is the target spectral peak of the signal and $\sum_{0}^{N}l^{2}(f)$ is the total energy of the spectrum of the signal. $f_{major}$ is the peak frequency and *B* = 0.1 Hz was the resolution in the estimation of the periodogram.

In summary, the purpose of this study was to remove the noise components in the radar signal and extract the respiratory and heart rate signals. To verify the de-noising effect, we analyzed the de-noised respiration and heart rate signals using the quantified indexes of *SNR* and *MSE*. The *SNR* characterizes the noise reduction effect and the *MSE* characterized the degree of difference from the ideal physiological signal. The larger the *SNR*, the better the de-noising effect. The smaller the *MSE*, the closer to the ideal signal. Therefore, it can be seen from the above Table 3 that the method in respiratory signal proposed in this paper had a higher *SNR*, as well as a lower *MSE*, as compared with the other two methods under the same noise intensity, and had a better noise reduction effect. The trend was consistent under different (0, 5, 10 dB *SNR*) conditions. Although EEMD-SampEn also reduced the noise, the residual noise was still present since the IMF component was still noisy; the de-noising effect was not good enough. Bandpass filtering had a higher *SNR* value for the de-noising of the respiratory signal, but the *MSE* value was very high and did not reflect the characteristics and details of the original signal. Heartbeat signals had a similar performance.

### 5.2. The Results of Measured Signal

**Figure 10:**
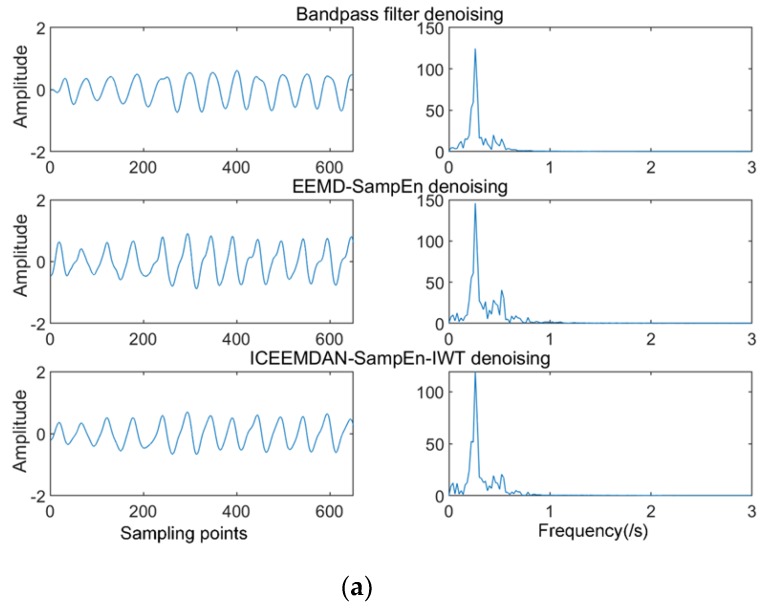

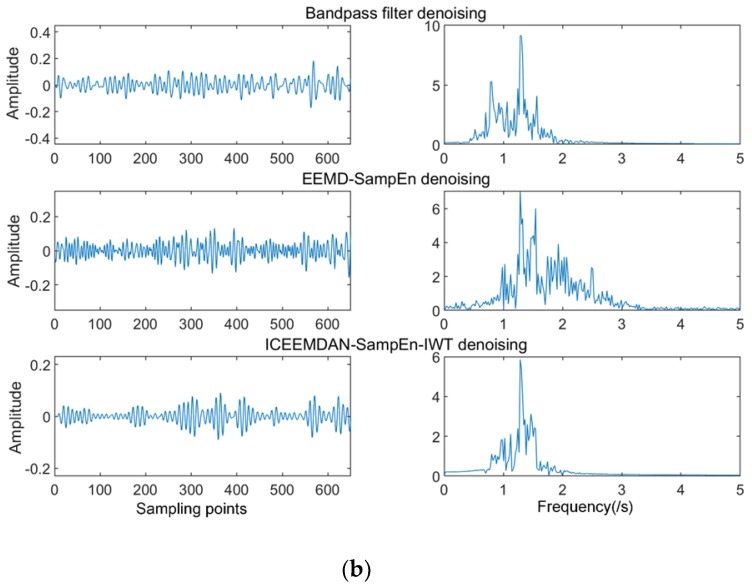
(**a**) Respiratory waveforms and spectrograms using three algorithms (measured signal). (**b**) Heartbeat waveforms and spectrograms using three algorithms (measured signal).

We collected a noise-containing radar signal with a duration of 60 s using a laboratory-made radar acquisition module. While collecting the radar signal, the Neulog devices were used to simultaneously collect the respiratory and ECG reference signals. Through spectral analysis, the primary frequency of the reference respiratory signal was 0.264 Hz, and the primary frequency of the reference heartbeat signal was 1.284 Hz. The ICEEMDAN-SampEn-IWT algorithm was proposed to de-noise the radar signal. As a comparison, we also used bandpass filtering and EEMD-SampEn to de-noise and separate the radar signal. The 50 s pre-processed radar signal and reference signal removed by the signal de-trending and the first and last invalid signals are shown in Figure 9. The de-noised respiration and heartbeat signals are shown in Figure 10, and the performance indicators are shown in Table 4.

Figure 9 and Figure 10 show that the proposed method had a more concentrated spectral peak. To further compare the performance of three typical algorithms, as can be seen from Table 4, the bandpass filtering de-noising method has a higher *SNR* when extracting the respiratory signal, and in other cases, it was not as good as the other two algorithms. The heartbeat signal extracted by the EEMD-SampEn method still had more noise signals. Comparing the three methods, the ICEEMDAN-SampEn-IWT algorithm performed best; both respiratory and heartbeat signals had the highest *SNR* and lowest *MSE* for this algorithm. The error in the above Table 4 was the major frequency difference between the de-noised signal and the reference signal. The algorithm proposed in this paper had the lowest estimation error. Although the error rates of the three methods were all below 1 BPM, the performance of the method used in this paper was the best and can be seen from the quantitative parameters closer to the ideal signal, such as the trend of exhalation and inhalation.

## 6. Conclusions

A novel radar vital signal separation and de-noising algorithm based on ICEEMDAN, sample entropy, and improved wavelet threshold was proposed. The ICEEMDAN was used to decompose the noisy radar signal into a series of IMF signals. By calculating the SampEn of each IMF, the noisy IMFs were screened and the improved wavelet threshold was used for de-noising. Then, spectrum analysis was performed on all the IMFs after de-noising. For the extraction of respiratory and heartbeat signals, the KL divergence values of each IMF were calculated, and appropriate IMFs were selected for signal reconstruction. Simulation and measured experimental results demonstrated the effectiveness of the algorithm. We also quantified the de-noising ability through two indicators, *SNR* and *MSE*. The results show that the algorithm had a better de-noising performance than other existing technologies. As a new de-noising algorithm, it effectively solves the problem of extracting accurate respiratory and heartbeat signals from noisy radar signals, especially for heartbeat signals, which are not easily extracted from respiratory harmonics and noise. This advanced technology is expected to be applied to health monitoring in the home. In future work, we will continue to improve the de-noising method of radar signals and explore the deployment of accurate vital signals in more complex noise backgrounds for deployment in the home environment.

## Figures and Tables

**Table 1:** SampEn value of each IMF.

IMFs	IMF1	IMF2	IMF3	IMF4	IMF5	IMF6	IMF7	IMF8	IMF9	IMF10
SampEn	1.644	0.7963	1.2	0.6754	0.2746	0.3751	0.2037	0.08601	0.02052	0.00746

**Table 2:** KL divergence value of each IMF.

IMFs	IMF1	IMF2	IMF3	IMF4	IMF5	IMF6	IMF7	IMF8	IMF9	IMF10
Kldiv	185.76	177.23	81.712	26.783	20.892	45.672	389.967	2167.8	5782.4	8532.6

**Table 3:** Performance indicators of respiration and heartbeat signals under different noise intensities and different de-noising algorithms.

*SNR*	Index	Denoising Method		
		Bandpass Filter	EMD-SampEn	ICEEMDAN-SampEn-IWT
Respiratory Signal				
0 dB	*SNR* (dB)	6.7562	2.8756	4.1224
	*MSE*	0.0825	0.0563	0.0347
5 dB	*SNR* (dB)	11.8184	7.2221	9.9485
	*MSE*	0.0658	0.0216	0.0142
10 dB	*SNR* (dB)	19.5873	12.8729	16.8541
	*MSE*	0.0412	0.0173	0.0087
Heartbeat Signal				
0 dB	*SNR* (dB)	−12.0412	−9.5428	−7.3873
	*MSE*	3.1159	1.8346	1.0342
5 dB	*SNR* (dB)	−10.5629	−8.7267	−4.0267
	*MSE*	1.9824	1.1785	0.8754
10 dB	*SNR* (dB)	−7.5673	−4.6734	0.2478
	*MSE*	0.9632	0.7382	0.0947

**Table 4:** Performance index and error estimation of respiration and heartbeat signals under different algorithms.

Types	Denoising Method			
	Index	Bandpass Filter	EMD-SampEn	ICEEMDAN-SampEn-IWT
Respiration	*SNR* (dB)	12.7826	9.8256	11.2174
	*MSE*	0.0842	0.0367	0.0173
	Error (BPM)	0.024	0.015	0
Heartbeat	*SNR* (dB)	−9.8341	−13.2675	−4.7712
	*MSE*	0.9173	0.3782	0.0958
	Error (BPM)	0.055	0.031	0.014
